# Unlocking genetic potential: a review of the role of CRISPR/Cas technologies in rapeseed improvement

**DOI:** 10.1007/s44154-025-00229-6

**Published:** 2025-05-07

**Authors:** Asif Mukhtiar, Saeed Ullah, Bo Yang, Yuan-Qing Jiang

**Affiliations:** State Key Laboratory of Crop Stress Resistance and High-Efficiency Production. College of Life Sciences, Northwest A & F University, Yangling, Shaanxi 712100 China

**Keywords:** Plant breeding, CRISPR/Cas, Crop improvement, Genome editing, *Brassica napus*

## Abstract

Rapeseed (*Brassica napus* L.) is a globally important oil crop, providing edible vegetable oil and other valuable sources for humans. Being an allotetraploid, rapeseed has a complex genome that has undergone whole-genome duplication, making molecular breeding rather difficult. Fortunately, clustered regularly interspacedshort palindromic repeat (CRISPR)/CRISPR-associated (Cas) technologies have emerged as a potent tool in plant breeding, providing unprecedented accuracy as well as effectiveness in genome editing. This review focuses on the application and progresses of CRISPR/Cas technologies in rapeseed. We discussed the principles and mechanisms of CRISPR/Cas systems focusing on their use in rapeseed improvement such as targeted gene knockout, gene editing and transcriptional regulation. Furthermore, we summarized the regulatory frameworks governing CRISPR-edited crops as well as the challenges and opportunities for their commercialization and adoption. The potential advantages of CRISPR-mediated traits in rapeseed such as increased yield, disease and stress resistance and oil quality are discussed along with biosafety and environmental implications. The purpose of this review is to provide insights into the transformative role of CRISPR/Cas technologies in rapeseed breeding and its potential to address global agricultural challenges while ensuring sustainable crop production.

## Introduction

Agriculture is facing mounting challenges due to escalating population of the world which is increasing with utmost great speed and predicted to increase by 25% in the next three decades reaching about 10 billion people(Hickey et al. 2019). In response to such remarkable challenges, deteriorating farmland and declining of field crop production, plant breeding techniques are considered to be the utmost need of the time to overcome such extraordinary challenges and enhance the improvement in field crops and ultimately the yield to feed such spreading population (Smil 2001). Various crop improvement techniques such as transgenic breeding, hybridization and mutation breeding have been employed and played a dominating role in agriculture. However, the introduction of specific alleles through hybridization or genetic recombination is time consuming and takes over several years (Scheben et al. 2017).

Genome editing techniques serve as the optimal tool for executing knockout, manipulating chromosomal recombination, and precisely inserting or substituting genetic bases at specific locations within the genes and chromosomes (Zhang et al. 2017). Prior to CRISPR/Cas discovery, genome editing was performed through DNA modification via site directed nucleases (SDNs), such as zinc finger nucleases (ZFNs) and transcriptional activator-like effector nucleases(TALENs) (Gaj et al. 2016). These systems enabled precise and specific gene modification subsidizing to improvement in crop productivity (Gupta et al. 2019). These two systems such as ZFNs and TALENs consisting of cleavage domain and DNA binding domain and dependent on endonucleases are efficiently engaged in field crops including wheat (Wang et al. 2014), tomato (Cermak et al. 2015) and maize (Shan et al. 2013). DNA binding domain of TALENs own high potential sequence when compared with that of ZFNs (Asmamaw and Zawdie 2021). Due to complexity of constructing these two systems, their application in plants at commercial scale is limited (Gaj et al. 2013; Liu et al. 2020a, b). Thus, these two techniques have been replaced with an advanced, more precise and convenient technology named as CRISPR (Woo et al. 2015). This system is based on clustered regularly interspaced short palindromic repeats (found in prokaryotes) acquired immune system to enhance defense mechanism in archaea and bacteria against the intruding plasmid and viral DNA which is based on endonuclease activity of protein (Cas) associated with CRISPR and is guided by crRNAs (Barrangou et al. 2007).

The CRISPR is considered to be a more efficient genome editing technique when compared with various SDNs, because of its high precision editing, which is due to specificity of guide RNA(gRNA) (Adli 2018).Genome editing, based on CRISPR is found to be useful for gene function identification in short time in new cultivars for field crop improvement. The CRISPR tool for genome editing gains advantage over past genome alteration techniques such as TALENs and ZFNs (Zhang et al. 2017). The prompt exploration of complex regulatory circuits is aided by the exploitation of multiple sgRNAs encoded within a singular CRISPR array, empowering the deletion of diverse target sites. In addition to deleting genes CRISPR is also used for introducing the DNA fragments in the target sites, and aids to amend the transcriptional activity specific to gene (Li et al. 2013; Lowder et al. 2015). After successful considerate the CRISPR mechanism in bacteria scientists moved a step forward to use this technique in humans and plants (Jinek et al. 2012).

The discovery by Doudna and Charpentier portrayed that any segment of DNA could be edited employing CRISPR/Cas9(Jinek et al. 2012). After its execution in 2013, this technology has been enormously applied to improve the genome of different field crops, such as soybean (*Glycine max*), tomato (*Solanum lycopersicum*), cotton (*Gossypium hirsutum*), rapeseed (*Brassica napus*), wheat (*Triticum aestivum*), sunflower (*Helianthus annuus*), rice (*Oryza sativa*) and many other ornamental and industrial crops (Ricroch et al. 2017; Liu et al. 2021). The use of CRISPR/Cas9 in the above crops proven that this method can contribute to research in area of plant functional genomics for improving crop productivity. CRISPR/Cas has been used in more than 50 plant species, including rapeseed, since its discovery (Metje-Sprink et al. 2019).

Various species of the Brassicaceae family, primarily *Brassica rapa*, *Brassica napus*, and *Brassica juncea*, which are cultivated for their oil or vegetables all over the world, have also employed this technique(Fard et al. 2018). The hybridization of two diploid species *(Brassica rapa* × *Brassica oleracea)* produced the allotetraploid species*Brassica napus,* which has a genome (AACC) and chromosomes (2n = 4x = 38)(Song et al. 2020).

Because allele sequences in *B. napus* are so similar, it is very challenging to study gene function. It is necessary to eliminate all homologous genes in order to produce consistent phenotypic traits in *B. napus* (Wells et al. 2014). CRISPR/Cas has helped to knock out multi-copy genes by confirming the ability to mutate multiple sites simultaneously (Wang et al. 2015). The primary factor impeding the improvement of rapeseed genetics is the B. *napus* single ancestral origin, which results in extremely low genetic diversity (Beilstein et al. 2006). Through various genetic techniques including hybridization and transgenic technology *B.napus* agronomics aspects have been greatly enhanced (Chen et al. 2019).

This work establishes a framework for comprehending gene mechanisms in diverse crops and their application in *B. napus* as well as the foundation for understanding past and present rapeseed breeding research including the use of CRSPR/Cas9 to knock out and silence distinct genes. The incredibly high genome editing accuracy of CRISPR/Cas has made it a potent tool in agricultural biotechnology, transforming traditional breeding methods and allowing the rapid domestication of wild species which speeds up crop improvement approaches. It also makes it possible to create novel crop species with desired traits (Puchta 2017). The use of CRISPR/Cas has greatly enhanced important agronomic traits, such as yield potential, nutrient quality, disease resistance, and herbicide tolerance. By precisely removing deleterious genetic factors for undesirable characters or improving gain-of-function mutations through targeted genome modification, CRISPR/Cas provides a reliable system for producing high-quality germplasm in comparison to traditional breeding techniques (Kumar and Jain 2015).This study also outlines the methods for determining which genes have been altered by CRISPR/Cas9 and which genes in *B. napus* can still be modified by researchers by thoroughly examining how altered genes contribute to genetic improvement.

## CRISPR classification and specifically the biology of CRISPR/Cas-9

**Fig. 1:**
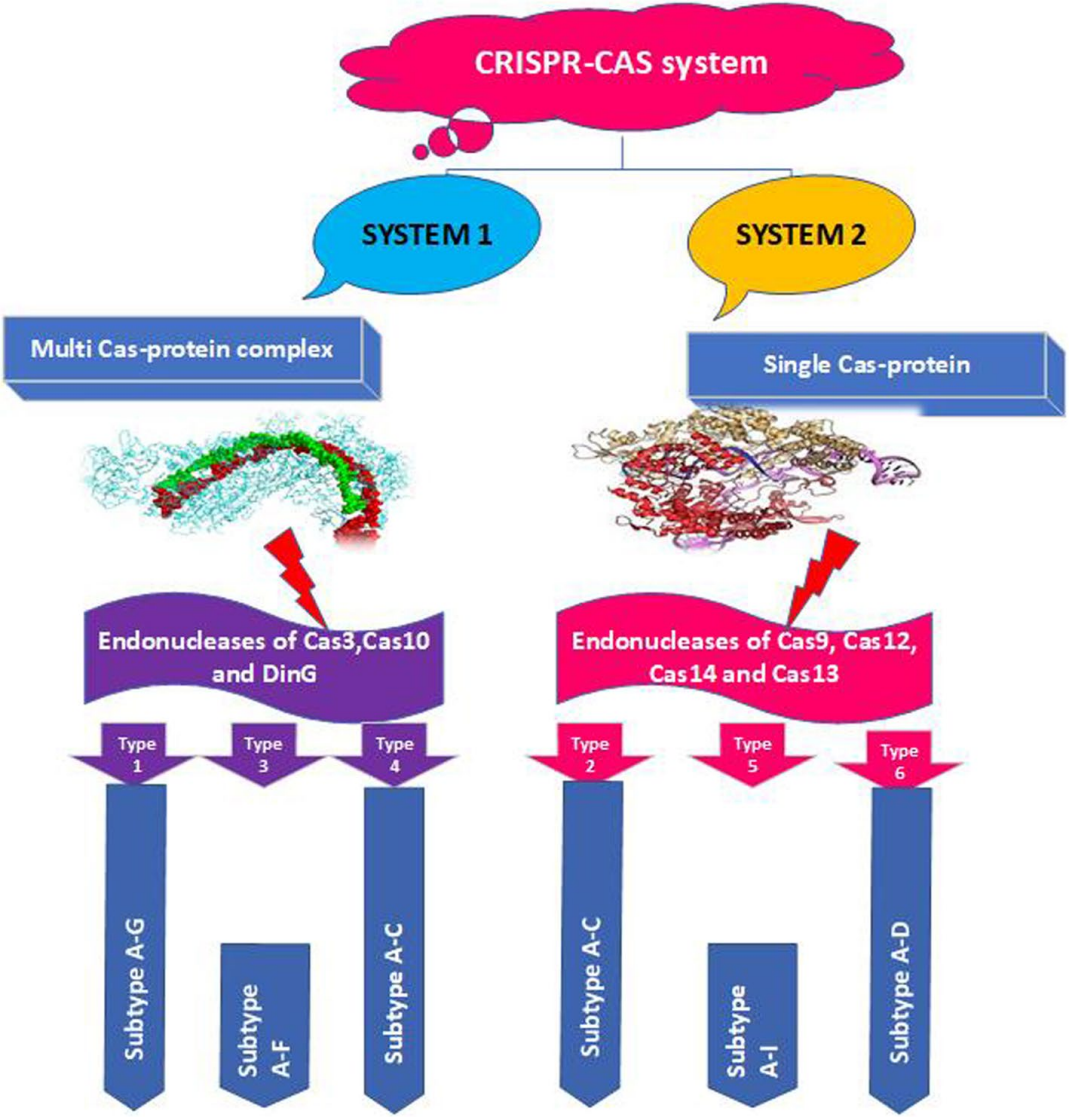
Illustrating the taxonomic classification of CRISPR based on the function of different systems. CRISPR-Cas system is categorized into two separate classes, distinguished by the design principles governing the effector modules. The system 1 encompasses various Cas proteins forming multisubunit structures, while system 2 possesses a single, extensive and multidomain protein as the effector

CRISPR-Cas system is categorized in two separate classes, distinguished by the design principles of their effector modules. The effector complexes of Class 1 systems encompass various Cas proteins forming multisubunit structures, while Class 2 systems possess a single, extensive and multidomain protein as the effector (Koonin and Makarova 2019) (Fig. 1). Because of its simple structure, the type II CRISPR/Cas-9 system is intensively analyzed and widely applied in the field of genetic engineering. The CRISPR/Cas-9 system consists of two core elements, which are the guide RNA (gRNA) and the CRISPR-associated protein (Cas-9). Initially, the Cas protein employed in gene modification was obtained from *Streptococcus pyogenes* and is known as SpCas-9. The CRISPR-associated protein Cas-9 is an extensive multi-domain DNA endonuclease with 1,368 amino acids. Its primary function is to cleave the target DNA and cause a double-stranded break (Liu et al. 2020a, b).

Cas-9 has two main regions which are the recognition (REC) lobe and other being nuclease (NUC) lobe. Within the REC lobe the domains, which are crucial for binding to gRNA includeREC1 and REC2, on the other hand, the interacting domains found in NUC lobe are Protospacer Adjacent Motif (PAM), RuvC and HNH domains(Liu et al. 2022). The two domains, RuvC and HNH, each cleave single-stranded DNA, while the PAM interacting domain ensures PAM specificity and initiates the binding process with the target DNA (Nishimasu et al. 2014).

The guide RNA consists of two elements such as trans-activating CRISPR RNA (tracrRNA) and CRISPR RNA (crRNA) (Faure et al. 2019). The crRNA which stretches 18–20 base pairs determines the target DNA by forming a specifically binding with DNA sequence (Newton et al. 2019). Conversely, tracrRNA comprised of series of extensive loops and serves to be a binding scaffold for the Cas-9 nuclease. Within prokaryotes, guide RNA targets viral DNA. In the context of genome editing toolsthe guide RNA could be artificially manufactured by integrating crRNA with tracrRNA resulting in a single guide RNA (sgRNA). This synthetic sgRNA is engineered to effectively target almost any gene sequence intended for editing (Liao and Beisel 2021).

## Mechanisms of CRISPR/Cas-mediated genome editing

**Fig. 2:**
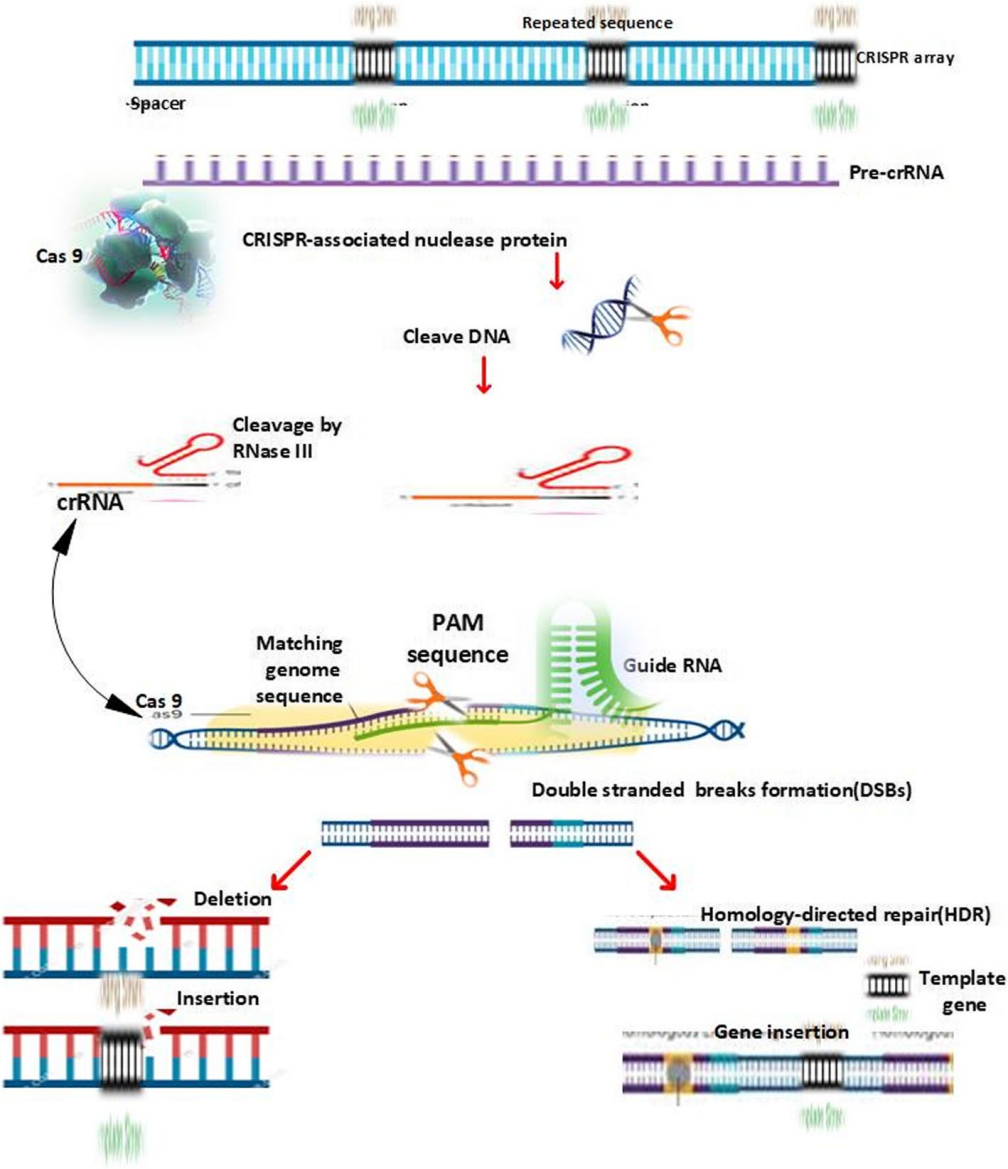
Showing the molecular mechanism of action of CRSIPR/Cas9. The Cas9 endonuclease,guided by the sequences on the single guide RNA (sgRNA) cleaves the targeted genomic sequence to create a blunt-ended double-stranded break (DSB) upstream of the protospacer adjacent motif (PAM).The presence of DSB triggers two endogenous DNA repair pathways, that is, non-homologous end joining (NHEJ, left side) and, homology-directed repair (HDR, right side).NHEJ is an error-prone pathway, in which two broken ends are simply religated, leading to uncontrolled insertions/deletions (InDels) at the junction of the rejoined chromosome

Based on DNA-RNA binding and recognition, CRISPR/Cas systems cause targeted DNA breaks acting as programmed genome modification tools (Jinek et al. 2012).They can be utilized for site specific targeted genomic sequences in DSBs. After DSB is made it activates the endogenous repair pathways such as NHEJ (non-homologous end joining) and HDR (homology-directed repair**)**. As NHEJ is a non-fidelity repair pathway, it results in unregulated insertions and deletions (indels) while repairing the DSB at the site of chromosome rejoining(van de Kooij et al. 2022). Natural chromosomal recombination is usually performed by homologous recombination during meiosis, which enables the exchange of genetic information accurately. CRISPR/Cas-mediated chromosomal rearrangements are optimally achieved by exploiting non-homologous end joining (NHEJ) mechanisms particularly in somatic tissues (Burma et al. 2006). NHEJ is one of the predominant DNA repair mechanisms and can be divided into two distinct pathways: classical NHEJ (cNHEJ) and alternative NHEJ (aNHEJ), which act in a partially antagonistic mode(Lieber et al. 2010). Targeted chromosomal rearrangements such as inversions, translocations and deletions may transpire between two DSBs that occur simultaneously (Chen et al. 2019) (Fig. 2).

Enhancing the precision of CRISPR-Cas9 technology with a focus on HDR efficiency optimization, has been of great interest in the context of applying gene therapy to human somatic cells. Homology-directed repair (HDR) is a versatile system for incorporating precise genetic modifications such as inserting protein epitope tags into genes, deleting genes, creating point mutations and altering enhancer and promoter sequences (Devkota 2018). For high-fidelity genome editing which minimizes unwanted mutations and off-target effects while precisely introducing desired genetic changes HDR efficiency must be increased. Improving the therapeutic potential of CRISPR-based gene editing particularly in clinical practice requires optimization of the CRISPR-Cas9 system which includes the use of engineered Cas9 variants, synthetic HDR enhancers and optimized delivery systems (Liao et al. 2024).

The Cas9 must bind to gRNA which can either be native crRNA or sgRNA, in order to recognize and cleave DNA at a specific location. The binding of sgRNA with Cas9 provides the best explanation of the arrangement of gRNA prior to target recognition (Jiang et al. 2015).The REC lobe encounters the most significant conformational change particularly with Hel-III, causing it to move approximately 65 angstroms toward the HNH domain when bound with sgRNA. On the other hand, Cas9 undergoes minor conformational changes on binding to target DNA and PAM sequence (Jinek et al. 2014).

Cas9 extensively interacts by directly binding sgRNA with the stem loop 1, repeat anti-repeat duplex and the linker region among stem loops 1 and 2. This interaction entails CTD domain Hel-I and the arginine-rich bridge helix (Jiang and Doudna 2017). Cas9 forms significant interactions with the ribose-phosphate backcrRNA-tracrRNA pair to form an active DNA surveillance complex (Jinek et al 2014). The principles governing the assembly of Cas9 and sgRNA bone of the gRNA arranging the 10-nt RNA sequence in an A-form conformation that is required for initial DNA interrogation (Künne et al. 2014). Upon binding to its gRNA, Cas9 forms a complex that is ready to search and identify complementary target DNA sites. Searching and identifying the target necessitate the alignment of the 20-nucleotide spacer sequence with a protospacer in the target DNA. Additionally, a conserved PAM sequence in close association with the target site must be present (Jiang et al. 2015).

## Importance of using CRISPR/Cas technologies in rapeseed

Rapeseed, an important oilseed crop holds a significant economic value and is widely used in various industries (Xiao et al. 2016). Currently, there is an imperative need of farming and production of rapeseed to improve agronomic traits using molecular genetics and genomics methods to modify the rapeseed genome. CRISPR/Cas9, being an innovative genome editing tool, is known for its ease and precision in genomic modification and has the potential to accelerate up the identification of gene functions and the development of new germplasm stock. After site-directed mutagenesis on two homologous genes (BnALC) usingCRISPR/Cas9 system for the first time in rapeseed in 2017, a transgenic T1 plant with four mutant alc alleles,generated from a single target sequence was successfully produced and passed down through subsequent generations with stable inheritance and no off-target effects (Braatz et al. 2017).

Researchers continue to worry about several potential drawbacks such as the challenges of deleting multi-copy genes and the potential for off-target effects. Rapeseed is an allotetraploid meaning that most of its genes have multiple copies with unnecessary functions making gene editing more difficult (Ihien Katche and S. Mason, 2023). Gene redundancy makes inducing trait changes through random mutations is extremely inefficient. Improving a single trait often involves the editing of several genes. Fortunately, using multiple single-guide RNAs (sgRNAs) makes it easier to direct the Cas9 protein to specific sites. This broadens up the possibility of performing multiple gene edits by employing Cas9 with the required sgRNA (Arora and Narula 2017).

The CRISPR/Cas9 technology encompasses distinct positive effects in polyploid rapeseed as it allows for the induction of multiple mutations in a single step. A common method in rapeseed is to induce modifications at multiple targeted sites at the same time by incorporating several independent cassettes expressing single-guide RNAs (Boniecka 2024). Currently, an increasing number of studies are focused on implementing the knockout of multi-copy gene in rapeseed through CRISPR/Cas9. These investigations highlight the promising potential of CRISPR/Cas9 in elucidating the functions of multicopy genes in rapeseed. The CRISPR/Cas9 is a highly effective and extensively adapted tool for high yield of crops whether using single or multiplex approaches of genome editing (Zhang et al. 2019).

Multiple gene mutants are frequently developed while studying the functions of homologous genes or members of genes families having high sequence similarity. According to several studies, various sgRNAs offer a significant advantage in modifying multiple genes due to their ability to precisely induce mutations in multiple genes (Feng et al. 2022). Typically, to increase mutation specificity, potential off-target sites must be verified. Researchers can use a variety of ways to minimize the probability of off-target mutations as much as it is feasible.

Recent research suggests that addressing off-target mutations can be accomplished through careful construction of specific single-guide RNAs (Du et al. 2023). As a result, designing sgRNAs with restricted off-target alterations is crucial for minimizing the possibility of unpredicted outcomes. Currently, in order to reduce off-target effects, researchers commonly use freely available online prediction tools to assist in the design of sgRNA (Sufyan et al. 2023). Furthermore, numerous approaches based on machine learning have been developed and implemented to identify sgRNAs with high on-target activity particularly in the field of agronomy(Liang et al. 2023).

The advancement of such methodologies aids to assess the action of designed sgRNAs in crops preventing unanticipated outcomes. This is also similar to Matres et al. (2021), which states that when gRNAs are carefully designed, off-target editing is minimal, with occurrence frequencies significantly lower when compared withthe natural diversity of plants. The primary limitation for breeders of rapeseed is not off-target mutations, because any unwanted genetic changes are expected to be rare and predictable (Walker et al. 2023). In brief, CRISPR/Cas9 has demonstrated the ability to concurrently modify several homologous genes without off-target edits producing stable mutations heritable in subsequent generations.

## Genes edited by CRISPR/Cas9 in rapeseed

There have been various genes modified by CRSPR/Cas 9 technique in different field crops for improvements of yield and resistant to various environmental and biotic stresses. The rapeseed is an allopolyploid and traits related to yield or stress tolerance are mainly controlled by alleles in A and/or C subgenomes making the necessity to knocking out several alleles. In this aspect traditional breeding is difficult for yield or stress improvement in rapeseed. This problem has not been solved using CRISPR/Cas9, which has revolutionized in the enhancement of production and combating to different stresses in rapeseed. Based on previous studies following are described some achievements made in rapeseed using the CRISPR/Cas9 technology.

### Yield-related attributes

Rapeseed yield is heavily influenced by factors such as seed size, seeds number per silique and quantity of siliques per plant and all of which are interrelated (Li et al. 2015). According to a study conducted by Khan et al. (2021), the average seed weight produced per plant was increased to 13.9% in quadruple mutants when compared with the wild type. This indicates that targeted mutants of *BnaEOD3* (*ENHANCER3 OF DA1*) copies perform redundant functions during development of seeds. Cytological observations suggested that *BnaEOD3* has a maternal role in stimulating the expansion and proliferation of cotyledon cells, which modulates seed growth in rapeseed (Khan et al. 2021). EOD3/CYP78A6 has also been demonstrated to increase the size of the seeds and the length of the siliques in Arabidopsis and wheat (Fang et al. 2012). Qi et al. (2019) found that sweet cherry fruit size decreased when PaCYP78A6 was suppressed through tobacco rattle virus-induced gene silencing (TRV-VIGS). This decrease was attributed to a reduction in mesocarp cell volume during development.

In order to modify both BnaBP genes in rapeseed, the researchers used the CRISPR/Cas9 system to create specific guide RNAs (sgRNAs). Individual knockouts of *BnaA03.BP* genes resulted in semi-dwarf and compact plant architecture, with no associated undesirable traits. As anticipated, plants with homozygous mutations in the *BnaA03.BP* gene exhibited a semi-dwarf stature (15.8%–16.9% shorter than the wild type) slightly drooping siliques and upright axillary buds (Fan et al. 2021).

Yield reduction in rapeseed commonly occurs from pod shattering during maturity constituting approximately 20% yield reduction and potentially escalating to 50% under extreme climatic conditions (Rasheed et al. 2021). Rapeseed is typically harvested before it reaches full maturity to reduce yield losses. However, this practice results in the extraction of oil from immature seeds, which contaminates the oil with chlorophyll and reduces its quality (Menendez et al. 2019). Furthermore, because pod shattering has a significant impact on the feasibility of mechanized rapeseed harvesting and thus improving pod shatter resistance is helpful to minimize the yield losses (Qing et al. 2021). Branch angle is another critical trait that influences planting density and the final yielding in rapeseed. Recently, through comparative transcriptomic analysis of 37 rapeseed accessions with divergent branch angle phenotypes followed by QTL-seq, *BnaWRKY40* was identified as being associated with branch angle trait and, RNAi silencing and CRISPR-mediated knockout of two *BnaWRKY40* alleles exhibited decreased branch angle (Sun et al., 2024). The above mentioned studies demonstrated that CRISPR/Cas 9 can be successfully applied as genome editing technique to modify the genome of rapeseed in order to enhance yield.

### Oil contents and fatty acids composition

Plant oils are important in agriculture because they provide a significant amount of edible oil for food processing and preparation. Enhancing seed oil content (SOC) and improving the composition of fatty acids (FAs) have consistently remained important objectives in rapeseed breeding (Beszterda and NogalaKałucka, 2019).

Yellow-seeded rapeseed has been the preferred choice for decades, due to its comparatively higher oil content, reduced pigmentation and lower fiber content compared with black-seeded varieties (Tian et al. 2022).The bHLH transcription factor TRANSPARENT TESTA (TT) 8 and the WDR protein TRANSPARENT TESTA GLABRA1 (TTG1) are essential for activating late genes involved in flavonoid biosynthesis (Patra et al. 2013). Allelic variation of *BnTT2* on the C genome has been shown to affect both seed color and fatty acid biosynthesis in rapeseed. The yellow seed trait is extremely beneficial, with the potential to improve seed quality and economic value. Xie et al. (2020)described how he used CRISPR/Cas9 technology to successfully generate stable mutants with yellow seeds. The researchers found that the *BnTT2* mutation has the potential to improve oil quality and yellow seed breeding implying a significant contribution to the economic value of rapeseed. Fatty Acid Desaturase-2 (FAD2) is an enzyme that plays a significant role in the production of plant polyunsaturated fatty acids. The yellow seed phenotype appeared only after removing all alleles from two BnTT2 homologues suggesting that BnTT2 homologues perform both conserved and redundant functions in seed color regulation. CRISPR/Cas9 technology was used to target multiple copies of the BnaFAD2 gene, resulting in new rapeseed varieties with improved fatty acid profiles (Huang et al. 2020).

### Nutritious and non-nutritious qualities

Improving nutrient resource utilization efficiency will result in lower fertilizer quantities, saving money on rapeseed production while also mitigating water eutrophication. Oilseed crops require a variety of macro- and micronutrients to function physiologically and reproductively. However, boron deficiency has a significant impact on oilseed rape yield and oil quality (Safdar et al. 2023). The soil contains 10 to 300 mg kg^−1^ of boron but only 5–10% is accessible to plants (Bhupenchandra et al. 2024).The primary effect of boron deficiency is the inhibition of root elongation. In the absence of sufficient boron, auxin redistribution occurs in the root elongation zone. This change in auxin content when combined with cytokinin and ethylene signals, inhibits root cell elongation (Chen et al. 2023).

Rapeseed displays hypersensitivity to boron deficiency, evident in the frequent occurrence of flowering without seed setting in the absence of sufficient boron (Rerkasem et al. 2020). In rapeseed, knockout lines of *BnaA9.WRKY47* developed using CRISPR/Cas9 demonstrated high sensitivity to low boron levels. These lines also exhibited lower boron contents compared with wild-type plants, as *BnaA9.WRKY47* is recognized for its strong binding activity with conserved sequences having a W-box in the promoters of B transport-related genes like *BnaBOR1s *(Tian et al. 2022).

Phytate also known as phytic acid (PA) is a compound containing phosphorus and produced due to the gradual phosphorylation of myo-inositol. It results in the formation of complexes with specific nutrient cations including Ca, Zn and Fe preventing their absorption. As a result, phytate acts as an anti-nutrient in the digestive tracts of humans. On the other side, PAs serve as a crucial storage form of phosphorus in seeds constituting up to 90% of the total seed content (Silva et al. 2021).Humans lack the enzyme phytase which is required for the metabolism of PA. As a result, consuming a large amount of PA reduces mineral and protein absorption by forming indigestible complexes with them (Shi et al. 2007). Consequently, researchers used CRISPR-Cas9 mutagenesis to silence functional paralogs of *BnITPK* yielding low PA mutants. This resulted in increased free phosphorus levels in canola (Sashidhar et al. 2020).

Rapeseed contains glucosinolates (GSLs) which are highly anti-nutritional along with other biologically active compounds. These substances account for a significant portion of the animal diet. Livestock consuming diets rich in glucosinolates experience detrimental effects such as diminished feed intake and growth, goiter, anemia, gastrointestinal irritation and the development of hepatic and renal lesions (Bischoff 2021).Within Brassica species, glucosinolates are conveyed from vegetative tissues to seeds through the action of glucosinolate transporters (GTRs) (Nour-Eldin et al. 2012). Researchers strengthened the rapeseed-specific transfection protocol. Using this refined method, they successfully edited the *BnGTR* genes, which regulate glucosinolate transport in rapeseed resulting in a high mutation frequency (Li et al. 2023a, b).

### Flowering time and flower development

The timing of flowering influences not only the yield of rapeseed but also the optimal sowing date for subsequent rotation crops. One of the main objectives of rapeseed breeding is to improve flowering time (Xu et al. 2016). Phosphatidylethanolamine-binding protein (PEBP) genes, belonging to the FLOWERING LOCUS T (FT)/TERMINAL FLOWER 1 (TFL1) family are crucial for plant development especially in regulating plant architecture and flowering timing (Freytes et al., 2021). After successfully disrupting four different homologous copies of TFL1 in rapeseed using CRISPR/Cas9 technology, researchers discovered that a specific mutation in BnaC3.TFL1 resulted in accelerated flowering (Sriboon et al. 2020). The*BnaA10.TFL1* controls rapeseed flower development by interacting with BnaA08.FD via the protein BnaA05.GF14nu, which leads to transcriptional repression of genes linked to floral integrator and floral meristem identity. Mutagenesis using CRISPR/Cas on up to four *BnTFL1* paralogs caused early flowering and changes to plant architecture (Wang et al. 2023).

Using CRISPR/Cas9 both SDG8 homologs,*BnaSDG8.A* and *BnaSDG8.C* were targeted for knockout in order to examine the impact on floral transition. The two mutant types subsequent analysis showed that BnaSDG8.A/C actively prevents the floral transition by directly increasing H3K36 m2/3 levels at the *BnaFLC* chromatin locus thereby playing a significant role in H3K36 m2/3 deposition (Jiang et al. 2018).

The successful generation of double mutants BnaA09.zep/BnaC09.zep resultedto orange flowers with a significantly higher lutein content and a markedly lower violaxanthin content. This study provides a valuable germplasm resource with orange-flowered traits for developing ornamental rapeseed varieties. Carotenoid biosynthesis genes and enzymes involved have also been extensively studied in a variety of plant species (Liu et al. 2020a, b). Another interesting study generated rapeseed plants with abscission-defective floral organs by simultaneously inactivating two *Inflorescence Deficient in Abscission* (*IDA*) genes using CRISPR/Cas9, thus lengthens the flowering duration for ornamental use (Wu et al., 2022). A more recent study demonstrated that CRISPR/Cas9-mediated knockout of rapeseed *Fruitfull* (*FUL*) gene, encoding an MADS-box transcription factor, caused delayed flowering providing a target for creating rapeseed germplasm with ideal flowering time (Min et al. 2024).

### Developing resistance to biotic and abiotic stresses

Biotic stresses, such as disease-causing microbes and weeds, as well as abiotic stresses like drought, salinity and cold have adversely affected the growth and yield of rapeseed. Inadequate crop rotation and tillage practices contribute to the accumulation of soil-borne pathogens which pose a significant threat to crop cultivation. The *Verticillium longisporum* is a hemibiotrophic soil borne pathogen that infects oilseed rape and Arabidopsis, causing verticillium stem striping in susceptible rapeseed. Researchers used transcriptome analysis to identify genes in rapeseed that were activated or up-regulated following Vl43 infection. The CRISPR-induced mutation in the *BnCRT1a* gene led to decreased susceptibility inrapeseed. Transcript analysis revealed that *CRT1a* loss of function activates the ethylene signaling pathway, which may contribute to the observed reduction in susceptibility (Pröbsting et al. 2020). Previous work as described in Table 1 shows that *WRKY11*and *WRKY70* regulate Arabidopsis disease resistance response to pathogens induced by salicylic acid (SA) and jasmonic acid (JA) (Jiang et al. 2016). Rapeseed production is frequently threatened by the necrotrophic pathogens *Sclerotinia sclerotiorum* and *Botrytis cinera*, causing the stem rot disease and the grey mould disease, respectively. A GWAS screening identified *RECEPTOR-LIKE KINASE 902* (*RLK902*) as a resistance gene and, knocking out *RLK902* by genome editing displayed a higher level of resistance to these two fungal pathogens (Zhao et al., 2024). More recently, it was reported that knockout of four homologous alleles of *BnaA07.MKK9* by CRISPR/Cas9 rendered more severe symptoms than the control when inoculated with necrotrophic fungus *S. sclerotiorum* (Lin et al., 2024).

**Table 1:** Depicting the various genes modified by CRISPR/Cas technologies in rapeseed

Target genes	Gene editing method	Phenotypic change	References
**Yield-related**			
* BnRGA, BnFUL, BnDA1, BnDA2*	CRISPR/Cas9 and multiple gene editin	Enhanced stem length	(Yang et al. 2017)
* BnCLV3*	CRISPR/Cas 9	Multilocular silique	(Yang et al. 2017)
* BnaBP* * BnaMAX1*	CRISPR/Cas 9	Plant architecture	(Zheng et al. 2020; Fan et al. 2021)
* BnEOD3*	CRISPR/Cas9 and multiple genome editing	Increased the weight of seed	(Khan et al. 2021)
* BnSHP1,BnSHP2* * BnJAG,BnALC,* * BnIND*	CRISPR/Cas 9	Pod shatter-resistant	(Zhai et al. 2019; Zaman et al. 2021)
* BnaA07.WRKY40.b, BnaC06.WRKY40.b*	CRISPR/Cas 9, RNAi	Decreased branch angle	(Sun et al., 2024)
**Oil content-related**			
* BnLPAT2 BnLPAT5* * BnTT8*	CRISPR/Cas 9	Seed color, oil content	(Zhang et al. 2019; Zhai et al. 2020)
* BnaFAD2*	CRISPR/Cas 9	Oleic acid content	(Huang et al. 2020)
* BnGTR2*	CRISPR/Cas9 and multiple gene editing	Seeds with small size, low glucosinolates and high oil contents	(Tan et al. 2022)
**Nutritious and non-nutritious qualities**			
* BnYCO*	CRISPR/Cas9 and multiple gene editing	Yellow cotyledon and chlorotic true leaves	(Liu et al. 2021)
* BnSPL3*	CRISPR/Cas9 and multiple gene editing	Delay in development	(Zhang 2018)
* BnTT2*	CRISPR/Cas 9	Yellow seeds	(Xie et al. 2020)
* BnTT8*	CRISPR/Cas 9	Yellow seeds	(Zhai et al. 2020)
* BnA9.WRKY47*	CRISPR/Cas9	Low boron sensitivity	(Feng et al. 2022)
**Flowering time and flower development**			
* BnAP2*	CRISPR/Cas 9	Flower development	(Zhang 2018)
* BnaSDG8*	CRISPR/Cas 9	Floral transition	(Jiang et al. 2018)
* BnMS5*	CRISPR/Cas9 and multiple gene editing	Male sterility	(Xin et al. 2020)
* BnA5.ZML1*	CRISPR/Cas 9	Reduction in self-compatibility	(Duan et al. 2020)
* BnaZEP*	CRISPR/Cas 9	Flower color	(Liu et al. 2020a, b)
* BnaTFL1*	CRISPR/Cas 9	Early flowering	(Sriboon et al. 2020)
* BnS6-SMI2*	CRISPR/Cas 9	Self-sterility	(Dou et al. 2021)
* BnYCO*	CRISPR/Cas9, multiple genome editing	Yellow cotyledon and having chlorotic true leaves	(Liu et al. 2021)
* BnaA03.BP*	CRISPR/Cas9, multiple gene editing	Semi- dwarfism with plant compact architecture	(Fan et al. 2021)
* BnC06.IDA, BnA07.IDA*	CRISPR/Cas9	abscission-defective floral organs	(Wu et al., 2022)
* BnaSVP*	CRISPR/Cas9 and multiple gene editing	Early-flowering	(Ahmar et al. 2022)
* BnaC09.FUL*	CRISPR/Cas9	Delayed flowering	(Min et al., 2024)
**Biotic and abiotic stresses**			
* BnWRKY70*	CRISPR/Cas 9	Sclerotinia-resistant	(Sun et al. 2018)
* BnaA07.MKK9*	CRISPR/Cas 9	Sclerotinia stem rot resistance	(Lin et al., 2024)
* BnaA05.RLK902*	CRISPR/Cas 9	Enhanced resistance to stem rot and grey mould diseases	(Zhao et al., 2024)
* BnaRGA*	CRISPR/Cas 9	Drought-resistant	(Yang et al. 2017)
* BnALS* * BnRGA and BnIAA7*	CRISPR/Cas 9, CBE	Herbicide-resistance	(Cheng et al. 2021)
* BnaA9.NF-YA7*	CRISPR/Cas 9	Drought tolerance	(Wang et al., 2024)
**Oil quality**			
* BnA10.LMI1*	CRISPR/Cas 9	Leaf shape	(Hu et al. 2018)
* BnD14*	CRISPR/Cas9, multiple gene editing	Dwarfism and prolific branched	Stanic et al. (2021)

There is a need for effective broad-spectrum herbicides to address herbicide resistance and weed population shifts in common cropping systems. Developing herbicide resistant varieties through breeding is a successful approach to manage weed stress (Biswas et al. 2023). Glyphosate is highly effective in destroying weeds, while causing minimal environmental harm (Ferrante et al. 2023). However, due to its toxicity to rapeseed it is not widely in rapeseed fields.. Mutations in the glyphosate-binding site of Enolpyruvyl Shikimate-3-Phosphate Synthase (EPSPS) genes have been shown to confer glyphosate resistance in a various plants (Dominguez-Valenzuela et al. 2023; Li et al. 2023a, b).

Drought-induced yield reduction poses a significant challenge that requires a thorough understanding of the various pathways and processes involved. Drought stress adversely impacts several aspects including germination, seedlings establishment, photosynthetic efficiency, mineral uptake, shoot elongation, seed development, and overall yield and quality (Batool et al. 2022). As a result, it is critical to investigate the physiological and molecular mechanisms governing rapeseed response to drought stress, particularly when breeding for drought resistance. According to Wu et al. (2020), BnaRGAs can interact physically with BnaA10.ABF2, a transcription factor involved in ABA signaling. BnaA10.ABF2 and BnaA6.RGA protein complexes significantly increased drought-responsive gene BnaC9.RAB18 expression. A more recent study identified the role of NUCLEAR FACTOR SUBUNIT A 7 (BnaNF-YA7) in drought tolerance in rapeseed, in which knockout and RNAi silencing of *BnaNF-YA7* caused higher survival rate by decreasing stomatal conductance and transpiration rate, compared with WT, while overexpression of this gene enhanced drought sensitivity (Wang et al., 2024). This suggests that BnaA9.NF-YA7 negatively regulates drought tolerance.

### Genes related to oil quality

Attaining superior quality traits is an importantaim in the breeding of oil crops. The rapeseed oil quality is predominantly influenced by factors such as its unsaturated fatty acid content, erucic acid levels and glucosinolate levels. To enhance the quality of edible oil, two main approaches have been identified by Gao et al. (2020), which involve augmenting the composition of both saturated and unsaturated fatty acids. In rapeseed, there exist two primary cellular biochemical pathways for unsaturated fatty acids. One of these pathways involves the transformation of unsaturated fatty acids into various unsaturated forms through the activity of the fatty acid desaturases (FADs).

The alternative pathway entails transforming unsaturated fatty acids into long-chain fatty acids exceeding 18 carbons, which is facilitated by the gene family named fatty acid elongases (FAEs). Concurrently, the targeted modification of specific controlling genes related to unsaturated fatty acids in rapeseed is anticipated to substantially boost the unsaturated fatty acid content. Editing multiple copies of the *FAD2* gene resulted in the successful development of individual plants exhibiting elevated oleic acid content (Okuzaki et al. 2018) (Table 1).This establishes a theoretical foundation for leveraging CRISPR/Cas9 genome modification technique to enhance components. Nevertheless, the executing this technique assists to modify the ratios of erucic acid and glucosinolate is advancing at a gradual pace and has not been documented.

## Conclusion and future perspectives

The use of CRISPR technology in rapeseed breeding is a significant advancement with profound consequences for agricultural sustainability and edible oil security. CRISPR has the potential to revolutionize rapeseed improvement by allowing precise and efficient genome editing, opening up opportunities for the development of novel traits and improving agronomic performance. The regulatory landscape for CRISPR-edited crops is changing, and as more countries develop clear guidelines and regulations, exploitation and adoption of CRISPR-edited rapeseed varieties are expected to accelerate.

Even though CRISPR/Cas has numerous advantages, including its unique capability to edit more than one genome, the system also has some crucial drawbacks and limitations. One of the most significant limitations is off-target mutagenesis, which vitiates the specificity and fidelity of genome editing and consequently limits its universal applicability. Furthermore, the overall reliance on stable transformation by *Agrobacterium tumefaciens* is a source of unpredictability in gene integration and is controlled by strict GMO laws, becoming a barrier to the commercialization of genetically edited crops in most of the world. In this context, development of transgene-free genome editing tools is essential to alleviate public concern (Zhang et al 2017). A virus-induced genome-editing protocol has been reported in wheat, and this approach could bypass tissue culture-based transformation (Li et al. 2021b). However, a similar approach waits to be developed for rapeseed.

Genome editing for agriculture has a very bright future with a potential to witness more of genetically engineered crops being commercialized. However, along with these, ethical and biosafety concerns need to be well addressed. Systematic risk assessment of potential impacts on human health, environmental balance, and non-target species is necessary as genome editing moves from containment in the laboratory to large-scale field use. Controls for mitigating unwanted genetic alterations must be scientifically validated and publicly documented to ensure maximum data confidence and regulatory effectiveness. For Brassica crops, CRISPR/Cas technologies will probably be a key tool in functional genomics and targeted breeding programs, particularly for seed oil composition modification and other agronomically desirable traits. With continuous advancements and optimized protocols, CRISPR/Cas will probably be a key tool in existing crop improvement strategies.

Looking into the future, more CRISPR technology research and innovation show promise for revealing more advantages in rapeseed breeding. Advances in multiplex genome editing, base editing and epigenome editing techniques may allow for simultaneous modification of multiple genes and regulatory elements, allowing for the rapid development of tailored rapeseed varieties with desired traits. Recently, a doubled haploid inducer-mediated genome-editing approach has been utilized to modify multiple gene homoeologs in rapeseed and *B.oleracea* (Li et al. 2021a), which can efficiently overcome the functional redundancy of homologous alleles of a gene in the allotetraploid of rapeseed. Integrating CRISPR technology with other breeding approaches, such as marker-assisted selection and genomic selection, could boost the efficiency and precision of rapeseed breeding pipelines. Addressing public acceptance and regulatory issues will be critical to the effective implementation of CRISPR-edited rapeseed varieties.

Investigating the potential applications of CRISPR technology beyond trait improvement, such as functional genomics, synthetic biology, and crop resilience to climate change may open up new avenues for increasing rapeseed productivity and resilience in the face of changing environmental and agronomic challenges. In conclusion, CRISPR technology is a transformative tool for rapeseed breeding, providing unprecedented precision and efficiency in genome editing. With continued research, innovation and stakeholder engagement, CRISPR-edited rapeseed varieties have the potential to significantly contribute to sustainable agriculture and global food and/or edible oil security in the coming years.

## Data Availability

The datasets generated and analyzedduring the current study are available from the corresponding author upon reasonable request.

## Abbreviations

ABE: Adenine base editor
CBE: Cytosine base editor
crRNAs: CRISPR RNAs
DSBs: Double-strand breaks
gRNA: Guide RNA CRISPR RNA (crRNA)
HDR: Homology-directed repair
NHEJ: Non-homologous end joining
SDNs: Site-directed nucleases
tracrRNA: Trans-activating CRISPR RNA
TALENs: Transcriptional activator-like effector nucleases
ZFNs: Zinc finger nucleases
